# Gut Microbiota and Cardiovascular Uremic Toxicities

**DOI:** 10.3390/toxins10070287

**Published:** 2018-07-11

**Authors:** Manuel T. Velasquez, Patricia Centron, Ian Barrows, Rama Dwivedi, Dominic S. Raj

**Affiliations:** 1 Division of Renal Diseases and Hypertension, The George Washington University, Washington, DC 20037, USA; mvelasquez@mfa.gwu.edu (M.T.V.); pcentron@mfa.gwu.edu (P.C.); rama.dwivedi@fda.hhs.gov (R.D.); 2 Department of Medicine, Georgetown University, Washington, DC 20007, USA; ianburrows@gmail.com; 3 United States Food and Drug Administration, Silver Spring, MD 20993, USA

**Keywords:** microbiota, microbiome, uremic toxicities, p-cresyl sulfate, indoles, trimethylamine-oxide, uremic toxins, intestinal microbiota, prebiotics, probiotics, synbiotics, adsorbents

## Abstract

Cardiovascular disease (CVD) remains a major cause of high morbidity and mortality in patients with chronic kidney disease (CKD). Numerous CVD risk factors in CKD patients have been described, but these do not fully explain the high pervasiveness of CVD or increased mortality rates in CKD patients. In CKD the loss of urinary excretory function results in the retention of various substances referred to as “uremic retention solutes”. Many of these molecules have been found to exert toxicity on virtually all organ systems of the human body, leading to the clinical syndrome of uremia. In recent years, an increasing body of evidence has been accumulated that suggests that uremic toxins may contribute to an increased cardiovascular disease (CVD) burden associated with CKD. This review examined the evidence from several clinical and experimental studies showing an association between uremic toxins and CVD. Special emphasis is addressed on emerging data linking gut microbiota with the production of uremic toxins and the development of CKD and CVD. The biological toxicity of some uremic toxins on the myocardium and the vasculature and their possible contribution to cardiovascular injury in uremia are also discussed. Finally, various therapeutic interventions that have been applied to effectively reduce uremic toxins in patients with CKD, including dietary modifications, use of prebiotics and/or probiotics, an oral intestinal sorbent that adsorbs uremic toxins and precursors, and innovative dialysis therapies targeting the protein-bound uremic toxins are also highlighted. Future studies are needed to determine whether these novel therapies to reduce or remove uremic toxins will reduce CVD and related cardiovascular events in the long-term in patients with chronic renal failure.

## 1. Introduction

Cardiovascular disease (CVD) remains a major cause of high morbidity and mortality in patients with chronic kidney disease (CKD) [1,2]. Risk factors, such as hypertension, diabetes, dyslipidemia, age, and coronary heart disease have traditionally been implicated in CVD in this population, but these factors do not entirely account for the high prevalence of CVD events or increased mortality rates in CKD patients [3,4]. Even in patients with advanced CKD or end-stage renal disease (ESRD), coronary events are responsible for a relatively small percentage of cardiac deaths, the most common causes are sudden death and heart failure [5].

In CKD, the loss of excretory function by the diseased kidney inevitably results in the retention of a variety of substances or solutes that are normally eliminated into the urine. This condition termed “uremia” refers to the disease resulting from renal failure that is unexplained by disturbances in extracellular volume, inorganic ion concentrations, or a decrease in renally synthesized substances [6]. Over the years, an increasing number of retained solutes, referred to as “uremic retention solutes”, have been identified, many of which have been found to be biologically active and exert toxicity that affect the functionality of virtually all organ systems in the body, leading to the clinical syndrome of uremia. Hence, these retention solutes are called “uremic toxins” [7,8]. In 2003, the European Uremic Toxin (EUTox) Work Group [7] classified 90 retention solutes into three major categories based on the molecular weight and kinetic behavior of the uremic retention solutes during dialysis:

(1) Small water-soluble molecules (molecular weight [MW] ≤ 500 Da), such as urea and phosphorus. Most toxins in this category are dialyzable with conventional dialysis. Some of them, such as urea and creatinine, have been used in the evaluation of renal excretory function and monitoring removal efficiency of dialysis treatment;
(2) Middle molecules (MW ≥ 500 Da). β_2_-microglobulin is a prototype of this group. Removal of middle molecules appears to be more effective with peritoneal dialysis than with conventional hemodialysis (HD). This is probably because of the larger pore sizes and the longer dialysis duration of peritoneal dialysis. Hemodialysis techniques that increased the permeability membranes (high-flux HD) or convection (hemofiltration/hemodiafiltration) provide superior clearance of these solutes, such as parathyroid hormone (PTH), β_2_-microglobulin, and advanced glycosylation end products.
(3) Protein-bound compounds, for example, 3-carboxy-4-methyl-5-propyl-2-furanpropionic acid (CMPF), indoxyl sulfate (IS), p-cresol sulfate (PCS), and homocysteine. Twenty-five of the 90 listed toxins (27.8%) are protein-bound, and 23 of them have an MW ≤ 500 D. Among these uremic toxins, organic anions, such as IS, CMPF, PCS, indole-3-acetic acid (IAA), and hippuric acid (HA) are low-molecular-weight compounds. Nevertheless, they should be classified as high-molecular-weight compounds in general circulation as they are firmly bound to plasma proteins, primarily albumin (molecular weight: 66 kDa). Therefore, this set of toxins is difficult to be dialyzed with conventional hemodialysis despite having molecular sizes small enough to pass through the dialysis membrane.

Subsequently, the list of uremic toxins has been expanded in a recent report by the EUTox Work Group, which described 32 previously known uremic toxins and 56 newly reported solutes, as well as their serum concentrations measured in uremic populations and healthy controls [9]. Notably, the variability in reported serum concentration of uremic toxins (as defined by the ratio between the highest and the lowest (H/L) concentrations) in uremic populations was found to be substantial for several protein-bound solutes (carboxymethyllysine, free IS, and phenol) and middle molecules (PTH, tumor necrosis factor (TNF)-α, leptin, osteocalcin, and interluekin-8) [9]. Moreover, the concentrations for a broad range of uremic toxins correlate poorly with estimated glomerular filtration rate (eGFR) [10].

## 2. Origin and Metabolism of Uremic Toxins

Uremic toxins may be viewed from the standpoint of their source or origin, production, and site (fate) of endogenous metabolism [11,12,13,14].

### 2.1. Urea

Urea, the prototype small molecule uremic solute is produced endogenously by the liver but undergoes further metabolism in the body and gastrointestinal tract [15,16]. In mammals and humans, urea is hydrolyzed in the alimentary tract [15]. The immediate end-product of the catabolism of proteins in mammals is ammonia. Ammonia is turned into urea via the ornithine cycle in order to decrease its toxicity, which is evident at higher concentrations. Mammals are not capable of metabolizing urea further into useful products and it is therefore considered to be a waste material removed via urinary excretion. Urea can also pass from the circulation into the gastrointestinal tract; here, urease-producing bacteria split urea into ammonia and carbon dioxide. Gut bacteria then utilize the ammonia as a source of nitrogen, producing amino acids and peptides necessary for growth. These microbial substances can be reabsorbed back into the host’s circulation and be utilized for synthetic processes, known as urea nitrogen salvaging [16].

### 2.2. Indole and Indole Components

Indole and indole derivatives are metabolites of tryptophan, an essential amino acid found in various foods, such as red meat, fish, and eggs, and are produced by intestinal bacterial degradation of tryptophan before absorption [17]. Indoles are metabolized to IS and indoxyl-β-d-glucuronide (IDG) in the liver [18]. Indole-3-acetic acid (IAA) is metabolized directly in the intestine [19] or in the tissue via tryptamine [20]. These tryptophan metabolites are endogenous ligands of the transcription factor aryl hydrocarbon receptor (AhR) that interacts with various regulatory and signaling proteins [21]. The activation of AhR mediates cardiotoxicity, vascular inflammation, and a procoagulant and prooxidant phenotype of vascular cells.

### 2.3. p-Cresyl Sulfate

PCS is a prototype protein-bound uremic toxin that originates in the intestine where gut bacteria metabolize aromatic amino acids, like tyrosine and phenylalanine, into phenolic metabolites, para-cresol, and 4-ethylphenol, which undergo sulfation by the liver, of which PCS is one of the components [22]. PCS levels have been shown to predict clinical outcomes in patients with CKD [23], and correlate with cardiovascular and all-cause mortality in CKD patients [24,25]. While PCS is not readily dialyzable by conventional dialysis, treatment with the oral adsorbent AST-120 [26], or with the prebiotic arabino-xylo-oligosaccharide [27], lowers plasma PCS levels. Vegetarians have been found to have lower levels of PCS than omnivores [28].

### 2.4. Trimethylamine N-Oxide (TMAO)

TMAO derives from dietary choline, where intestinal bacteria metabolize choline to trimethylamine (TMA), a gas that is then absorbed into the circulation and subsequently oxidized to TMAO by hepatic flavin-containing monooxygenases (FMOs) [29]. The intestinal microbiota metabolism of dietary l-carnitine, a source of TMA abundantly found in red meat, was also shown to produce TMAO and accelerates atherosclerosis [30].

TMAO seems to add to the advancement of atherosclerosis in part by promoting cholesterol accumulation within macrophages, possibly by inducing scavenger receptors like CD36 and SRA1, both of which are involved in the uptake of modified lipoproteins [31]. Fasting levels of TMAO in plasma predict the risk of major adverse cardiovascular events independently of traditional cardiovascular risk factors and the existence or amount of coronary artery disease [32].

## 3. Altered Gut Microbiota in Chronic Kidney Disease

The gut microbiome, comprising of the collective genomes of the communities of commensal microbes (microbiota) that colonize the gut, is involved in numerous physiological processes, including nutrient extraction/synthesis, metabolism, and immune regulation [33,34,35,36,37]. Alterations in the composition and function of intestinal microbiota, also referred to as “gut dysbiosis”, is characterized frequently by decreased diversity, and the relative abundance of selected microbial taxa in the intestine [38]. Recent evidence has emerged that the gut microbiota composition is altered in CKD. For example, Vaziri et al. [39], in their studies of humans and animals, found significant differences in the abundance of 190 microbial operational taxonomic units (OTUs) between patients with end-stage renal disease (ESRD) and normal control individuals. The microbial families showing the largest increase in ESRD patients were from *the Actinobacteria*, *Firmicutes (especially subphylum Clostridia)*, *and Proteobacteria (primarily Gammaproteobacteria)* phyla. In addition, studies in rats with CRF induced by 5/6 nephrectomy and in control rats also showed a significant difference in the abundance of 175 bacterial OTUs between the uremic and control animals, most notably as decreases in the *Lactobacillaceae and Prevotellaceae* families [39]. Furthermore, ESRD patients exhibited a significant expansion of bacterial families possessing urease, uricase, and indole and p-cresol forming enzymes, and contraction of families possessing butyrate-forming enzymes [40].

A number of factors contribute to dysbiosis in patients with CKD/ESRD include a slow intestinal transit [41], impaired protein assimilation [42], decreased dietary fiber intake [43], iron therapy [44], and frequent use of antibiotics [45,46].

## 4. Altered Gut Microbiota in Cardiovascular Disease

Altered gut microbiota composition has also been reported in CVD [47,48]. In a metagenome-wide association study in fecal samples of 405 Chinese subjects comprising of 218 individuals with atherosclerotic cardiovascular disease (ACVD) and 187 control subjects, Jie et al. [47] reported that the gut microbiome of ACVD individuals differed from that of healthy controls by having an increased abundance of *Enterobacteriaceae and Streptococcus* spp. Further analysis showed that the gut microbiome function from stool samples of ACVD subjects also differed functionally from that of healthy controls, notably in the potential for metabolism or transport of several molecules important for cardiovascular health. For example, the gut microbiome of those with ACVD had a higher potential for the transport of simple sugars (phosphotransferase systems (PTS)) and amino acids, and for the metabolism of glycerolipids and degradation of fatty acids. Interestingly, gut microbial enzymes involved in the formation of TMA, the precursor for the proatherogenic metabolite TMAO, were enriched in the ACVD samples compared to healthy controls. 

In another a study in Sweden, Karlsson et al. [48], using shotgun sequencing of the gut metagenome, found that the genus *Collinsella* was enriched in patients with symptomatic atherosclerosis (defined as stenotic atherosclerotic plaques in the carotid artery leading to cerebrovascular events), whereas *Roseburia* and *Eubacterium* were enriched in healthy controls. Analysis of the functional capacity of the metagenomes showed that genes encoding peptidoglycan synthesis were enriched in ACVD patients, whereas genes involved in synthesis of anti-inflammatory molecules (such as butyrate) and antioxidants were enriched in control subjects. These findings suggest that the gut metagenome may contribute to the development of symptomatic atherosclerosis by acting as a regulator of host inflammatory pathways.

## 5. Gut microbiota and Uremic Toxins

Almost 50 years ago, Mitch [49] called attention to the importance of intestinal bacteria on nitrogen metabolism in CKD patients. In nitrogen balance studies performed in uremic patients before and during oral administration of aminoglycoside antibiotics, he found that nitrogen derived from urea is not used by uremic patients for amino acid synthesis and that the negative nitrogen balance in these patients significantly improved during the antibiotic treatment. Based on these observations, he suggested that intestinal bacteria adversely affect CKD patients by promoting catabolism and by producing toxins which accumulate in body fluids. More recently, Aronov et al [17] assessed the contribution of the colon to the production of several uremic solutes in hemodialysis (HD) patients and showed that IS and PCS were almost absent in HD patients without colons, suggesting that colonic microbes may produce an important portion of uremic solutes, which may contribute to uremic illness.

A number of studies, both in humans and animals, have provided further evidence that altered gut microbiota could contribute to the increased production of gut-derived uremic toxins in CKD [50,51,52].

Barrios et al. [50] analyzed metabolite associations with 16S gut microbiome profiles in a large cohort of 855 individuals with early kidney disease. They found that phenylacetylglutamine was associated with 52 Operational Taxonomic Units (OTUs), indoxyl sulfate with three OTUs, and PCS with only one OTU. All microorganisms pertain to the order *Clostridiales*, represented by the *Christensenellaceae*, *Ruminococcaceae* and *Lachnospiraceae* families, and among them, three were associated with renal function as well. These data suggest that indoxyl sulfate, p-cresyl-sulfate, and phenylacetylglutamine are early markers of deterioration of renal function. Changes in the intestinal flora associated with these metabolites are measurable in early kidney disease. 

The impact of gut microbiota on uremic toxins has also been investigated in animal models with experimental renal failure [51,52]. For example, Mishima et al. [51], using capillary electrophoresis time-of-flight mass spectrometry (CE-TOFMS), performed a comprehensive analysis of uremic solute profiles (in plasma, feces, and urine) in adenine-induced renal failure (RF) and control mice under germ-free (GF) or specific pathogen-free (SPF) conditions. Important changes in plasma metabolites were noted in mice with renal failure under GF conditions. Of the 183 solutes found, plasma levels of 11 solutes were significantly lower in GF mice than in SPF mice with renal failure. These 11 solutes were thought to be microbiota-derived uremic solutes and included IS, PCS, phenyl sulfate, cholate, hippurate, dimethylglycine, γ-guanidinobutyrate, glutarate, 2-hydroxypentanoate, TMAO, and phenaceturate. Germ-free renal failure (GF-RF) conditions resulted in the disappearance of colonic short-chain fatty acids (SCFAs) and decreased utilization of intestinal amino acids, but paradoxically they showed worse renal disease in comparison with the renal failure SPF mice. SCFAs produced by gut bacteria and the effective use of amino acids may be protective for the kidneys, and a decrease in these factors may exacerbate renal damage in germ-free mice with renal failure. These findings suggest microbiota contribute substantially to the production of toxic uremic compounds, but conversely, growth without microbiota has harmful effects on CKD progression. The probable protective effects on renal function of microbiota may be in part due to the effective use of dietary amino acids and colonic SCFA production, since a reduced intestinal use of dietary amino acids and the absence of SCFAs in the colon was observed on germ-free mice.

Kikuchi et al. [52] analyzed uremic toxin production and the composition of gut microbiota in CKD rats and cecectomized rats and observed that serum and urine levels of IS and phenyl sulfate were higher in CKD versus control rats. Administration of AST-120, a spherical carbon adsorbent, decreased uremic toxin production and changed the global composition of intestinal microbiota in CKD rats. UT urinary excretion and intestinal microbial composition changed in cecectomized rats, with *Clostridia*-affiliated species found in abundance and *Bacteroidia*-affiliated species found to be greatly decreased (*p* < 0.01). Identified candidate indole- and phenol-producing intestinal microbiota, 3 *Clostridia*, and 2 *Bacteroidia*. These OTUs have a tryptophanase/tyrosine phenol-lyase gene in the closest-sequenced genome out of the OTUs that declined following cecectomy. These data indicate that UT production is correlated with a subset of indigenous gut microbiota.

Collectively, these studies indicate that changes in gut microbiota in CKD contribute importantly to the increased production of uremic toxins observed in renal failure.

## 6. Uremic Toxins and Cardiovascular Disease

In recent years, it has become increasingly evident that uremic toxins play an important role in cardiovascular damage associated with CKD. Several clinical studies in CKD and non-CKD patents have shown an association between uremic toxins and CVD [53,54,55,56,57,58,59,60,61,62,63,64,65]. For example, Sato et al. [53] measured plasma IS levels in patients with known CAD and eGFR averaging 60 mL/min/1.73 m^2^. Using echocardiography, they observed that in patients with a higher total IS levels, there was a higher proportion of left ventricular dysfunction compared to patients with lower levels. Shimazu et al. [54] found an increased risk of hospital admissions for heart failure and cardiac death in early CKD patients with cardiomyopathies and higher levels of total indoxyl sulfate.

IS has also been associated with higher degrees of coronary artery calcification and cardiac drug-eluting stent re-stenosis [55,56]. Lin et al. [57] reported that higher total indoxyl sulfate levels were associated with an increased risk of cardiovascular events but not mortality in patients with CKD stage 3 to 5. A meta-analysis of 11 studies (10 prospective studies and one cross-sectional study) with a total of 1572 patients with stage 1–5 CKD showed that increased levels of PCS are correlated with both increased risk of CVD events and mortality in CKD patients, while elevated levels of IS were found to be correlated with increased mortality, but not with CVD events [58]. However, data are mixed in HD patients. One study showed an association of IS with heart failure among patients on HD [59]. Other studies failed to demonstrate the association of IS levels with cardiovascular mortality [60,61,62,63]. In a prospective study in 499 patients with mild-to-moderate kidney disease, Meijers et al. [64] reported that higher baseline concentrations of free PCS were directly associated with cardiovascular events, an association that was independent of GFR and Framingham risk factors. Another prospective cohort study of 112 elderly hemodialysis patients showed that serum-free PCS was associated with all-cause and CVD mortality beyond traditional and uremia related risk factors [65].

There are also studies showing an association of uremic toxins with vascular disease. In a cohort study of 199 patients at different stages of CKD, Barreto et al. [66] were the first to demonstrate that since very early in the development of CKD, there is a gradual rise in serum levels of IS, which correlates with the severity of CKD. Higher serum levels of indoxyl sulfate were also correlated with aortic calcification, vascular stiffness, and with increased risk of all cause and cardiovascular mortality not depending on age, sex, diabetes mellitus, phosphorus, albumin and hemoglobin levels, vascular stiffness, or aortic calcification.

In a cross-sectional study of 149 CKD patients, Rossi et al. [67] reported that serum-free and total IS were independently associated with serum levels of certain inflammatory markers, and PCS (free and total) were separately associated with interleukin-6 and pulse-wave velocity. Free IS and PCS were both also separately correlated with plasma glutathione peroxidase [GPx] activity, a marker of oxidative stress.

Similarly, in a prospective study of 120 patients with CKD, Dou et al. [68] demonstrated that serum IAA was an important predictor of mortality and CVD events after adjusting for age, gender, cholesterol, systolic and diastolic BP, smoking, CRP, phosphorus, BMI, albumin, history of CVD, PCS, and IS, and remarkably, after adjusting for CKD stages as well. In addition, serum IAA levels were directly related to markers of inflammation and oxidative stress, namely C-reactive protein and malondialdehyde, respectively. Moreover, in vitro studies using cultured human endothelial cells showed that by triggering an inflammatory nongenomic aryl hydrocarbon receptor pathway, IAA produced cyclooxygenase-2, a proinflammatory enzyme. It also triggered endothelial inflammation and oxidative stress [68].

In another prospective study of 821 consecutive patients with peripheral artery disease (PAD), it was shown that elevated TMAO levels were associated with 2.7-fold increased all-cause mortality risk [69]. Moreover, mortality risks were not significantly different among all different subtypes of diagnosis of peripheral arterial disease, presence or absence of coronary artery disease, as well as other clinical and laboratory parameters. A relationship between serum TMAO levels with early atherosclerosis has recently been reported in a recent study of 220 subjects who participated in the Tübingen Lifestyle Intervention Program [70]. In this study, fasting serum TMAO levels were positively correlated with carotid intima-media thickness (cIMT), an early marker of atherosclerosis. Higher TMAO levels predicted increased cIMT, independently of age, sex, and visceral fat mass, and other cardiovascular risk markers. Interestingly, during a standard lifestyle intervention, mean cIMT decreased significantly in subjects in the tertile that had the largest decrease of TMAO levels (>20%).

## 7. Effects of Uremic Toxins on the Myocardium

In vitro studies in cultured cardiac myocytes have shown that perfusion with culture media containing 20 mM urea and combined with creatinine (5 mM) plus urea (200 mM) reduced energy charge (calculated from the ratio of [ATP], [ADP], and [AMP]) [71]. This effect was more evident after administration of an artificial uremic medium (comprised of uremic serum, urea, creatinine, and cytokines). The cardiodepressive effect of uremic serum (2.5%) was entirely reversed by the calcium agonist, Bay K 8644, demonstrating disruption in myocardial calcium homeostasis in uremia. As contractility of myocytes is reduced due to administration of uremic toxins or uremic serum, the changes in myocardial contraction frequency or inotropy was attributed to dysregulation of calcium availability within the cell.

Studies in cultured cardiomyocytes have shown that p-Cresol decreased the spontaneous contraction rates of cardiomyocytes, caused irregular cardiomyocyte beating, and induced structural and functional changes in the gap junction [72]. Moreover, p-cresol increased intracellular Ca(^2+^) levels, and induced Ca(^2+^)-dependent protein kinase Cα (PKCα) activation. P-cresol-induced gap junction disassembly appears to be mediated by PKCα, since these effects were prevented by a PKCα inhibitor or SiRNA knockdown of PKCα.

In studies done in hypertensive rats, IS was found to augment oxidative stress and decrease anti-oxidative defense, which in turn was associated with a worsening of cardiac fibrosis and cardiomyocyte hypertrophy [73]. In cultured neonatal rat cardiomyocytes, indoxyl sulfate also induced cardiomyocyte hypertrophy and increased ROS levels in a time and dose-dependent manner [74]. These effects were associated with the inhibition of AMP-activated protein kinase (AMPK) enzymatic action and reduced expression of uncoupling protein 2 (UCP2). Cardiomyocyte hypertrophy and down-regulation of UCP2 by IS was appropriately reduced when pretreated with an AMPK activator, suggesting that inhibition of AMPK/UCP2 signaling and the increase of oxidative stress may have played a role in the IS induced cardiomyocytes hypertrophy.

Studies in a CKD rat model induced by 5/6-nephrectomy (5/6-STNx) showed that increased serum IS levels were associated with the development of diastolic dysfunction [75]. The STNx animals developed cardiac hypertrophy and fibrosis, correlated with increased expression of cardiac proteins, such as TGF-β, phospho−NF-κB, phospho-p44/42, and phospho-p38, as well as gene expression of profibrotic and hypertrophic markers. Treatment with AST-120 significantly reduced serum IS levels, improved renal function, and markedly reduced cardiac fibrosis. 

Recent work suggests that increased ambient TMAO concentration may alter mitochondrial energy metabolism in the myocardium [76]. Both LEAK (substrate-dependent) and OXPHOS (oxidative phosphorylation-dependent) mitochondrial respiration with pyruvate were reduced when cardiac fibers were acutely exposed to TMAO. Another important finding was the impaired substrate flux via pyruvate dehydrogenase. Administering TMAO at 120 mg/kg for 8 weeks resulted in increased plasma and cardiac tissue concentrations by a factor of 22–23. Longstanding TMAO administration reduced mitochondrial LEAK state respiration with pyruvate by 30% with no effect on OXPHOS state respiration. In addition, both long-term TMAO administration and acute exposure to TMAO reduced respiration with palmitoyl-CoA indicating deranged β-oxidation. Taken together, these indicate that elevated levels of trimethylamine *N*-oxide hinders pyruvate and fatty acid oxidation in heart mitochondria.

## 8. Effects of Uremic Toxins on the Vasculature

Experimental studies have shown that uremic toxins exert direct effects on the vasculature [77,78,79,80,81,82,83,84,85,86]. For example, urea at concentrations seen in chronic renal failure was shown to induce mitochondrial ROS production in cultured primary human aortic endothelial cells (HAEC) [77], resulting in deleterious cellular effects, such as the direct inactivation of the enzyme PGI2 synthase, an anti-atherosclerosis enzyme, endoplasmic reticulum stress, activation of intracellular pro-inflammatory pathways, and accumulation of intracellular advanced glycation end products (AGEs). Furthermore, atherosclerosis from uremia can be averted by treating uremic mice with an SOD/catalase mimetic.

Another potential toxic effect of urea is through carbamylation of proteins and amino acids [78]. At high concentrations in uremia, urea can transform spontaneously to cyanate. The highly reactive form of cyanate, isocyanic acid, induces nonenzymatic modification or carbamylation reaction of proteins, amino acids, and other molecules, changing their structure and function. This modification is considered an adverse reaction and has been linked to inflammation, atherogenesis, and CVD [79,80], and was shown to be predictive of mortality in ESRD patients [81]. 

Protein-bound uremic solutes, PCS and IS have been shown to inhibit endothelial proliferation of human umbilical vein endothelial cells (HUVEC) in-vitro [82]. IS was also shown to induce oxidative stress by increasing the production of reactive oxygen species (ROS) and NAD(P)H oxidase activity, and decreasing glutathione levels in HUVEC [83]. IS directly stimulates proliferation of rat vascular smoot muscle cells and activates mitogen-activated protein kinase (MAPK) in vitro [84]. In an in vivo rat model using intravital microscopy, exposure to uremic levels of IS, PCS, and p-cresyl glucuronide (PCG) induced pronounced effects on the recruitment of circulating leukocytes in the peritoneal vascular bed [85]. Superfusion with IS resulted in the induction of strong leukocyte adhesion, increased diapedesis, and restricted circulation; furthermore PCS resulted in the augmentation of white blood cell rolling. Superfusion with PCS and PCG combined resulted in deranged circulation and extravasation, and it did not augment white blood cell rolling more than PCS alone. Moreover, intravenous infusion with IS confirmed the superfusion results. These findings demonstrate that IS, PCS, and PCG induce pro-inflammatory substances that lead to vessel injury by promoting the interaction between white blood cells and blood vessels. 

Finally, TMAO was shown to induce inflammation with the release of inflammatory cytokines IL-1β and IL-18 and triggers oxidative stress with the inhibition of endothelial nitric oxide synthase (eNOS) nitric oxide (NO) production in HUVECs [86]. These effects appear to be mediated via activation of ROS-TXNIP-NLRP3 inflammasome, suggesting a possible mechanism for TMAO enhancement of atherosclerosis and inflammation.

**Figure 1:**
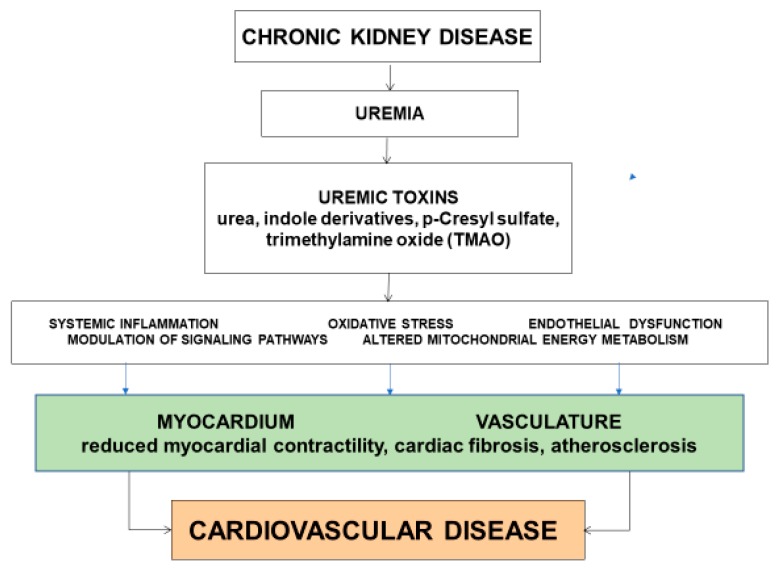
Putative mechanisms linking uremic toxins to the development of cardiovascular disease.

The underlying mechanisms or pathways that link uremic toxins to the development of CVD are complex and incompletely understood. Several mechanisms have been proposed, including systemic low-grade inflammation, oxidative stress, endothelial dysfunction, modulation of signaling pathways, and altered mitochondrial energy metabolism (Figure 1).

## 9. Therapeutic Interventions

Dietary therapy is an integral part of the management of patients with chronic renal failure. Changes in quantity and quality are made in order to provide the nutrients needed to obtain homeostasis, and for correcting nutritional and metabolic imbalances, reducing retained toxic substances that are responsible for the disease process, and preserving residual renal function. Dietary protein restriction is a well-established intervention to prevent or reduce the symptoms of uremia in CKD patients [87,88]. Since diets rich in protein, such as animal meat, poultry, etc., are also rich sources of many uremic toxins, it is not surprising that a low-protein diet can improve symptoms of uremia [88].

### 9.1. Prebiotics

Prebiotics are a group of non-digestible carbohydrates that selectively change both the constitution and the activity of the microbiome in a way that benefits the host [89]. Prebiotic foods, such as dietary fibers, different oligo- and polysaccharides, and resistant starches, are increasingly recognized as beneficial for maintaining a healthy gut microbiota [90]. To date, there are few studies that have investigated the effects of prebiotics in animals and humans with CKD. Vaziri and co-workers have shown that a high amylose-resistant starch diet retards renal disease progression and attenuates oxidative stress and inflammation in rats with experimental CKD [91]. In subsequent studies in rats with adenine-induced CKD, these investigators further showed that a high amylose maize-resistant starch type 2 diet (HAMRS2) diet decreased cecal pH and microbial diversity, and increased the *Bacteroidetes-to-Firmicutes* ratio [92]. Levels of serum and urine indoxyl sulfate, as well as urine p-cresol, were reduced in HAMRS2-fed rats.

One clinical study in CKD patients reported consuming foods with added fiber (23 g/day fiber daily) for 4 weeks results in significant decreases in BUN and serum creatinine and improvement in eGFR [93]. In an open-label study in maintenance HD patients, Meijers et al. [94] showed that supplementation with the prebiotic oligofructose-enriched inulin for 4 weeks produced significant reductions in generation rates and serum concentrations of PCS. Sirich et al. [95] compared the effect of resistant starch (amylose) versus digestible starch (amylopectin) supplementation for 6 weeks in patients on HD and found a significant reduction in serum IS, and a non-significant reduction of PCS in the resistant starch-treated group, but not in the amylopectin-treated group. Taken together, these studies suggest that prebiotics have favorable effects in CKD with reductions in some gut microbiota-derived uremic toxins and improvement in renal function. Additional studies are needed to study the long-term effects of prebiotics on uremic toxins and CVD outcomes in patients with CKD.

### 9.2. Probiotics

Probiotics refer to live microorganisms with the ability to improve the health of a host when given in appropriate quantities. There are only a few clinical trials that have tested the effects of probiotics on CKD. In a randomized placebo-controlled crossover study involving 22 hemodialysis patients, administration of Renadyl (*Lactobacilli acidophilus*, *Bifidobacteria longum*, and *Streptococcus thermophiles*) did not produce significant change in serum levels of uremic toxins or markers of inflammation and oxidative stress [96]. In another randomized controlled trial conducted in 39 peritoneal dialysis patients, Wang et al. [97] found a significant reduction in the serum levels of endotoxin and pro-inflammatory cytokines, increased levels of interleukin-10 (an anti-inflammatory agent), and preservation of residual renal function after administration of probiotics for 6 months. Oral treatment of eight HD patients with *L. acidophilus* for up to 6 months showed decreased serum dimethylamine and nitroso dimethylamine, two of the uremic toxins associated with increased mortality in CKD [98]. Oral treatment with *B. longum* has been shown to lower IS levels in HD patients [98,99] and slow progress of CKD and *S. thermophiles*, which are mainly present in fermented food, such as yogurt, and by processing high urease activity, has been shown to significantly lower the serum urea following supplementation of *S. thermophiles* in food of uremic rats [100].

### 9.3. Synbiotics

The rationale for synbiotic therapy is that the prebiotic will nourish the organisms administered as a probiotic and facilitate their engraftment and proliferation. Guida et al. [101] conducted a randomized placebo-controlled intervention trial of a combination of a probiotic and prebiotic (synbiotic) in 30 patients with CKD (stages 3–4) and found that treatment with Probinul neutro^®^, a synbiotic that normalizes intestinal microflora, for 4 weeks resulted in a significant reduction in the total plasma PCS concentrations. Another randomized controlled trial by Rossi et al. [102] found that synbiotic therapy for 6 weeks effectively lowered serum concentrations of PCS, and to a lesser extent, IS in patients with moderate to severe CKD. In this study, the synbiotic regimen changed the composition of the fecal microbiome, namely increasing *Bifidobacterium* and diminishing *Ruminococcaceae*.

### 9.4. Adsorbant AST-120

AST-120 is an oral intestinal sorbent that promotes the fecal excretion of uremic toxins and precursors by binding to them in the gastrointestinal tract. It effectively binds to indoles, decreasing both the serum and urinary levels of IS [103]. AST-120 has been available outside the United States in countries like Japan (since 1991), Korea (since 2005), and the Philippines (since 2010), and is mainly used to reduce uremic symptoms and prolong the time to initiation of hemodialysis. Both carotid artery intima and media width, and pulse-wave velocity were decreased in patients with nondiabetic CKD treated with AST-120 for a year compared with those who did not receive the intestinal sorbent [104]. In patients with congestive heart failure and moderate CKD (serum creatinine 1.3–2.0 mg/dL), treatment with AST-120 at a dosage of 6 g/day, in addition to conventional therapy for 24 months, improved signs and symptoms of heart failure, serum atrial natriuretic peptide, and renal function, and decreased the number and length of hospitalizations [105]. However, in a large randomized controlled trial, long-term use of AST-120 for 36 months in CKD patients with advanced CKD (stage 3 or 4) added to standard treatment did not produce greater reductions in the concentrations of uremic toxins (i.e., serum and urine IS, and serum *β*2-miroglobulin), renal disease progression, proteinuria, mortality, and hospitalizations [106].

### 9.5. Genetically Engineered Bacteria

Microbes can be engineered into “smart” living therapeutic agents programed to produce a continuous and inexpensive supply of heterologous molecules of biomedical interest that could be used in the treatment of myriad of diseases. Urea and ammonia removal using microencapsulated, genetically engineered *Escherichia coli* DH5 cells has been described. When given orally to uremic rats, during their passage through the intestine, small molecules like urea diffuse rapidly into the microcapsules and are acted on by the genetically engineered cells, which lower plasma urea and ammonia levels [107]. Bacterial tryptophanases convert tryptophan to indole, which is absorbed and modified by the host to produce IS. In vitro studies showed that deleting this gene eliminates the production of indole in vitro. A majority of individuals in the genus *Bacteroides* have the most abundant tryptophanases in their gut. In animal models, by altering the gene expression of tryptophanse or reducing the abundance of the *Bacteroides* they were able to reduce the generation of IS [108]. Certain methanogenic archaea could use TMA as their substrate for growth. Preliminary studies from our lab show that gut colonization with specific methanogenic archaea reduced TMAO levels and attenuated atherosclerosis (Unpublished observation).

### 9.6. Dialysis Modalities

Conventional HD treatment efficiently removes urea and other small size and unbound toxic solutes that readily pass through capillaries and membranes. Removal of other uremic toxins may be limited due to large molecular size, protein binding, or sequestration within body compartments. Recent studies suggest that better outcomes on morbidity and mortality can be accomplished by increasing the removal of uremic toxins in the molecular weight category; this can be attained with high-flux membranes and more effective hemodialysis methods [109,110]. For example, in a randomized controlled trial involving a large number (*n* = 456) of chronic HD patients, Maduell et al. [109] showed that switching to high-efficiency post-dilution online hemodiafiltration (HDF) reduced all-cause mortality by 30% compared to regular HD. A pooled individual data analysis from four randomized controlled trials showed that online HDF compared with conventional HD reduces the risk of mortality in ESRD patients [110].

Recently, novel medium cut-off (MCO) dialysis membranes have been developed to better assimilate the natural kidneys with larger pores and with improved pore size distribution and permeability [111]. Benefits of MCO dialyzers include their ability to remove more uremic toxins than conventional high-flux (HF) membranes and their ability to retain albumin by forming a protein layer covering the synthetic membrane during dialysis, which restricts the removal of molecules with a radius over 3.5 nm, resulting in the optimization of large uremic toxin removal.

In two recent pilot studies, the effects of HD therapy using an MCO dialyzer (MCO HD) was compared to (a) high-flux HD, and (b) high-flux HD and HDF [112]. The primary outcome was the clearance of overall free immunoglobulin light chains (lambda FLC) while the clearance of other middle molecules and safety was the secondary outcome. In these two studies, MCO HD resulted in superior clearance of lambda free light chains compared with high-flux HD and HDF. These findings suggest that MCO HD is better than high-flux HD and HDF in terms of their ability to remove larger middle molecules.

A recent randomized crossover trial in 48 chronic dialysis patients showed that MCO dialysis for 4 weeks led to enduring changes in plasma levels of many cytokines and other molecules, such as a reduction in sTNF-R1, kappa and lambda free light chains and urea, as well as a rise in LpPLA2 compared to HF dialysis [113]. These results suggest that MCO dialysis could possibly be used to improve the clearance of middle molecules in maintenance dialysis patients. Additional studies using MCO dialyzers with longer treatment periods are needed to confirm these findings.

### 9.7. Peritoneal Dialysis

The transport of solutes and water does not utilize artificial membranes in peritoneal dialysis (PD); it occurs between the peritoneal cavity and the blood in the surrounding capillaries found in the peritoneal membrane. Small solutes are transported across the peritoneal membrane mainly by diffusion, whereas transport of larger molecules in the parietal is dominated by convective forces [114]. Net ultrafiltration depends on the opposing forces of the osmotic and hydrostatic pressures in the cavity.

Previous studies have shown that the total and peritoneal clearance of urea nitrogen and creatinine are remarkably higher than the clearances of beta-2-microglobulin, p-cresol sulfate, and phosphate in patients on PD [115]. This is consistent with the diffusive and convective transport through the pores of the peritoneal membrane [116,117]. Eloot et al. [118] compared the effects of automated PD (APD) versus continuous ambulatory peritoneal dialysis (CAPD) on the total clearance and mass removal of different uremic toxins in patients on PD patients and found that total clearance and mass removal of small and water-soluble molecules were higher for CAPD compared with APD; this is thought to be due to a better residual renal function (i.e., higher urine output in CAPD) with no difference in the peritoneal contribution. Drained volume and obtained ultrafiltration were increased with APD versus CAPD, but no differences in clearance or mass removal were observed for the protein-bound solutes. Even though the drained dialysate volume was significantly increased to almost double, both APD and CAPD showed similar peritoneal clearance and solute removal. Additionally, APD showed superior ultrafiltration, but worse residual urinary output and clearance.

## 10. Summary and Conclusions

Humans host a diverse community of microbes, which colonize the skin, oral cavity, airways, and gastrointestinal tract. They exhibit complex interactions with the host and other microbiota in the community. The purpose of natural selection on the microbiota is not to benefit the host; rather, microbiota evolution is dominated by the need for each species to compete with other community microbiota for survival. It is possible that a uremic environment fosters the growth of specific microbiota that generate uremic toxins as a metabolic byproduct. While removing the uremic toxins alone is a temporizing measure, the possibility of establishing symbiosis is the holy grail of treatment investigators are ardently pursuing. The advances in DNA technologies for manipulation of microbial genomes, combined with the increasing knowledge of the molecular basis of diseases, are promoting the development of engineered smart bacteria, and is paving the way for precision microbiome-based treatment of diseases.
